# Cocreating a Harmonized Living Lab for Big Data–Driven Hybrid Persona Development: Protocol for Cocreating, Testing, and Seeking Consensus

**DOI:** 10.2196/34567

**Published:** 2022-01-06

**Authors:** Teemu Santonen, Despoina Petsani, Mikko Julin, Markus Garschall, Johannes Kropf, Vicky Van der Auwera, Sylvie Bernaerts, Raquel Losada, Rosa Almeida, Jokin Garatea, Idoia Muñoz, Eniko Nagy, Eva Kehayia, Elaine de Guise, Sylvie Nadeau, Nancy Azevedo, Sofia Segkouli, Ioulietta Lazarou, Vasileia Petronikolou, Panagiotis Bamidis, Evdokimos Konstantinidis

**Affiliations:** 1 Department of Research, Development, Innovation and Business Development Laurea University of Applied Sciences Espoo Finland; 2 Laboratory of Medical Physics and Digital Innovation, School of Medicine, Aristotle University of Thessaloniki Thessalonki Greece; 3 Center for Technology Experience AIT Austrian Institute of Technology Vienna Austria; 4 Salumentis OG Vienna Austria; 5 LiCalab, Thomas More University of Applied Sciences Geel Belgium; 6 Expertise Unit Psychology, Technology & Society, Thomas More University of Applied Sciences Antwerp Belgium; 7 Fundación INTRAS, Research, Development and Innovation Projects Department, Spain Valladolid Spain; 8 GAIA, Asociación de Industrias de Conocimiento y Tecnologías Aplicadas Basque Country Spain; 9 Nagykovácsi Wellbeing Living Lab Nagykovácsi Hungary; 10 Centre for Interdisciplinary Research in Rehabilitation of Greater Montreal (CRIR) Montreal, QC Canada; 11 School of Physical and Occupational Therapy, McGill University Montreal, QC Canada; 12 Department of Psychology, Université de Montréal Montreal, QC Canada; 13 School of Rehabilitation, Université de Montréal Montreal, QC Canada; 14 Centre for Research and Technology-Hellas (CERTH)/Information Technologies Institute (ITI) Thessaloniki Greece; 15 European Network of Living Labs Brussels Belgium

**Keywords:** Living Lab, everyday living, technology, big data, harmonization, personas, small-scale real-life testing, mobile phone

## Abstract

**Background:**

Living Labs are user-centered, open innovation ecosystems based on a systematic user cocreation approach, which integrates research and innovation processes in real-life communities and settings. The Horizon 2020 Project VITALISE (Virtual Health and Wellbeing Living Lab Infrastructure) unites 19 partners across 11 countries. The project aims to harmonize Living Lab procedures and enable effective and convenient transnational and virtual access to key European health and well-being research infrastructures, which are governed by Living Labs. The VITALISE consortium will conduct joint research activities in the fields included in the care pathway of patients: rehabilitation, transitional care, and everyday living environments for older adults. This protocol focuses on health and well-being research in everyday living environments.

**Objective:**

The main aim of this study is to cocreate and test a harmonized research protocol for developing big data–driven hybrid persona, which are hypothetical user archetypes created to represent a user community. In addition, the use and applicability of innovative technologies will be investigated in the context of various everyday living and Living Lab environments.

**Methods:**

In phase 1, surveys and structured interviews will be used to identify the most suitable Living Lab methods, tools, and instruments for health-related research among VITALISE project Living Labs (N=10). A series of web-based cocreation workshops and iterative cowriting processes will be applied to define the initial protocols. In phase 2, five small-scale case studies will be conducted to test the cocreated research protocols in various real-life everyday living settings and Living Lab infrastructures. In phase 3, a cross-case analysis grounded on semistructured interviews will be conducted to identify the challenges and benefits of using the proposed research protocols. Furthermore, a series of cocreation workshops and the consensus seeking Delphi study process will be conducted in parallel to cocreate and validate the acceptance of the defined harmonized research protocols among wider Living Lab communities.

**Results:**

As of September 30, 2021, project deliverables *Ethics and safety manual and Living lab standard version 1* have been submitted to the European Commission review process. The study will be finished by March 2024.

**Conclusions:**

The outcome of this research will lead to harmonized procedures and protocols in the context of big data–driven hybrid persona development among health and well-being Living Labs in Europe and beyond. Harmonized protocols enable Living Labs to exploit similar research protocols, devices, hardware, and software for interventions and complex data collection purposes. Economies of scale and improved use of resources will speed up and improve research quality and offer novel possibilities for open data sharing, multidisciplinary research, and comparative studies beyond current practices. Case studies will also provide novel insights for implementing innovative technologies in the context of everyday Living Lab research.

**International Registered Report Identifier (IRRID):**

DERR1-10.2196/34567

## Introduction

### Virtual Health and Wellbeing Living Lab Infrastructure Project in Brief

The Horizon 2020 Project VITALISE (Virtual Health and Wellbeing Living Lab Infrastructure), funded by the European Union (EU; under grant agreement 101007990; April 2021-March 2024), unites 19 partners across 11 countries. VITALISE aims to harmonize Living Lab procedures and enable effective and convenient transnational and virtual access to key European health and well-being research infrastructures (RIs), which are governed by Living Labs. To do so, the VITALISE project itself follows a Living Lab procedure grounded on agile, user-centric, and multistakeholder-driven cocreation and testing approaches. A series of joint research activities (JRAs) in the fields of (1) rehabilitation, (2) transitional care, and (3) everyday living environments act as testing arenas for cocreating and validating the harmonized Living Lab procedures. Thematically, the JRA research protocol described in this study focuses on health and well-being research in everyday living environments among older adults.

### Living Labs as an RI

The European Network of Living Labs (ENoLL), which is an international nonprofit association promoting and enhancing user-driven innovation ecosystems and is also the coordinator of VITALISE project, defines Living Labs as “user-centred, open innovation ecosystems based on systematic user cocreation approach, integrating research and innovation processes in real life communities and settings” [1]. A systematic literature review explored the key characteristics of the Living Lab approach and concluded that the definitions for Living Labs and their use vary significantly in the literature, whereas some common elements can be identified [2]. The Living Lab harmonization work conducted in the context of 15 health and well-being Living Labs across the Baltic Sea region acts as a starting point for this study [3]. In VITALISE, Living Labs are considered RIs. In regulation 1291/2013, the EU Parliament and Council of the EU define RIs as “facilities, resources and services that are used by the research communities to conduct research and foster innovation in their fields” [4]. RIs can be (1) a single-sited facility (ie, unified single body of equipment at 1 physical location), (2) distributed facility (ie, a network of distributed resources such as instrumentation, collections, archives, and scientific libraries), (3) web-based facility (ie, information and communications technology–based systems for scientific research), and (4) mobile facility (ie, vehicles design for scientific research) [5]. The everyday living environments in JRA case studies comprise (1) public outdoor (eg, natural parks and outdoor fitness areas) and indoor (eg, museum and sports hall) places and (2) private homes, day care facilities for older adults, and smart home Living Labs equipped with sensors and other technological devices.

### Big Data as Dynamic Persona Enabler

Big data have been described as the *future of health care* as it may generate (1) better evidence for health care delivery; (2) improve data quality and access; (3) help drive better communication among patients, providers, and communities; (4) identify trends; and (5) provide better information for patients and authorities [6,7]. In the context of Living Lab–driven research and innovation activities, multiple opportunities for using big data approaches can be identified, including (1) comparative effectiveness research to determine clinically relevant and cost-effective ways to diagnose and treat patients; (2) identify needs, follow-on indications, and adverse effects, which otherwise would stay hidden; and (3) use real-time data for safety monitoring and event prediction [8].

Personas—hypothetical user archetypes created to represent a user community—have been argued to be a powerful technique in the early design process phases to define and prioritize requirements and user needs [9]. Nevertheless, no databases exist that represent the aging and chronic patient population in a holistic and deep way, making it difficult to create effective personas [10]. The 3 primary ways to define personas include (1) qualitative personas, (2) quantitative personas, and (3) qualitative personas with quantitative validation, also known as hybrid personas [11,12]. Prior studies have identified various challenges regarding the creation and distribution of personas, including (1) the lack of knowledge of the technique, (2) lack of resources (time and funding), (3) sparse descriptions relying on too much on qualitative data, (4) small sample size, and (5) lack of first-hand user and patient data [13,14].

Owing to increasing popularity of big data and digitalization-driven opportunities, data-driven persona development has been suggested as a solution to create more reliable personas [12]. Therefore, VITALISE case studies focusing on everyday living environments adopt the hybrid big data approach and use various state-of-the-art technologies to collect real-time big data on top of the user-provided qualitative data. The possible technologies in case studies include, for example, serious games; mobile phone apps; user behavior monitoring with 3D depth sensors and red, green, and blue wavelength cameras; and sensor data. As each Living Lab and case study also have their own research priorities, there is a need to effectively manage big data collection to enable open data sharing between different Living Labs, which is one of the main outcome objectives of the VITALISE project. Prior studies have argued that a top-down approach is needed to achieve the effective management of big data [6]. Therefore, the proposed efforts regarding a harmonized research protocol for developing big data–driven hybrid personas have a justified foundation.

### Objectives

The objectives of this study are described next. First, the aim is to collectively identify the most suitable methods, tools, and instruments to collect qualitative and quantitative data (ie, following a mixed methods approach) [15] among Living Labs in the context of big data–supported everyday living environment research. Second, by following triangulation principles [16], a group of small-scale case studies focusing on everyday living environment interventions will be conducted to evaluate the applicability of the selected methods, tools, and instruments in various real-life Living Lab environments, covering (1) applied- and solution-oriented and (2) use-inspired basic research settings [17]. Each case study intervention has specific research objectives, which are defined more specifically in the *Case Study Interventions for Phase 2* section. Third, a cross-case analysis regarding the challenges and benefits of using the proposed technologies, methods, tools, and instruments will be conducted to cocreate and define a harmonized research protocol for developing big data–driven hybrid personas. The aim is to reach a consensus regarding defined research protocols among the case study Living Labs and external harmonization body, which is a wider community of stakeholders consisting of Living Lab researchers and practitioners, health care professionals, and policy makers.

## Methods

### Research Process Phases

#### Overview

A solution-oriented research paradigm covering (1) ex ante and engaging (ie, research activities before the case studies), (2) in situ and engaging (ie, research activities during the case studies), and (3) ex post and engaging (ie, research activities after the case studies) phases will be used as the overall theoretical framework for this study [18]. Among scholars and practitioners, there is no clear consensus on what are and how many stages there are in Living Lab research and innovation processes [19]. The different multistaged iterative Living Lab processes have very similar activities; however, the naming for the different phases varies [3]. By combining 2 prior studies’ suggestions regarding Living Lab research process phases [3,20], the data collection phases for this study are given in the following sections.

#### Phase 1: The Ex Ante and Engaging

Phase 1 focuses on exploring the problem space by collectively identifying the most suitable Living Lab methods, tools, and instruments for health-related research conducted in everyday living environments [21,22]. The following research questions (RQs) have been defined for this phase:

- RQ1: What are the most potential data and big data collection technologies for health and well-being Living Lab research in the context of everyday living environment research?
- RQ2: What are the most potential methods, tools, and validated scientific instruments for health and well-being Living Lab research in the context of everyday living environment research?

On the basis of identified needs and opportunities, a joint research protocol will be cocreated to cover the interplay among the case studies and enable open data sharing among the case studies.

#### Phase 2: The In Situ and Engaging

Phase 2 focuses on experimentation by conducting a group of small-scale case studies in various Living Labs and everyday living environment interventions and settings, as presented in Table 1.

**Table 1:** Case study key interventions and data collection settings.

Case studies and key interventions			Virtual or auditive interaction		Sensor technology
**Case study 1: Combination of distributed facility (participants homes) and virtual reality environments (ie, mobile phone coaching app)**					
	An 8-week training period to maintain social relationships and activities that could be at risk by the withdrawal from the workplace	Virtual coaching via mobile phone app		—^a^	
**Case study 2: Single-sited facility (smart home Living Lab) equipped with virtual reality environments (** **Windows-based software** **)**					
	Series of cognitive and physical exercises, including 1-hour yoga exercise	Measurement of financial capacity (banking game) and cognitive status (virtual supermarket game) as well as physical activity promotion via virtual coach by using app		Wristband sensors (Fitbit Charge 3 and Empatica E4) during all activities and Muse EEG^b^ (InterAxon Inc) during the yoga exercise	
**Case study 3: Single-sited facility (measurement laboratory) equipped with virtual reality environments (** **Windows-based software** **)**					
	A total of 2 weekly sessions of 30 minutes during the 8 weeks training period focusing on physical and cognitive training	Windows-based software having combined physical and cognitive training (dual task) exercises		Motion capture via Kinect v2 (Microsoft)	
**Case study 4: Distributed facilities (sport halls, nursing homes, and outdoor training facilities) with mobile facilities (wearable sensors)**					
	Comprehensive physical fitness evaluation in a multistaged measurement track and recreational football playing (Spain) and outdoor fitness trail training (Hungary) for the 8 weeks period	In the case of COVID-19 restrictions, YouTube and WhatsApp group video calls will be used for virtual exercise coaching in Spain		Wearable activity sensors (SenseWear Armband [BodyMedia Inc] and Galaxy Watch 3 [Samsung Electronics])	
**Case study 5: Distributed facilities (museums) with various mobile facilities (wearable sensors and trackers)**					
	Explore the lived experience of the museum visit with the help of innovative technologies	Description of the museum exhibit items, including the audio version of the written information that accompanies the work and adaptive version of the information for people with disabilities		Eye-tracking glasses (Pupil Labs), smartwatch (Apple Watch or Fitbit inspire2), NIRS^c^ system (Artinis Medical System), pressure sensitive walkway (Gaitrite, GAITRite), and APDM inertial sensors (APDM Wearable Technologies Inc)	

^a^Sensor technology has not been used.

^b^EEG: electroencelogram.

^c^NIRS: near-infrared spectroscopy.

As a result, *data triangulation* using multiple data sources, *investigator triangulation* using >1 investigator, *theory triangulation* using multiple theoretical approaches to interpret the phenomenon, and *methodological triangulation* using multiple quantitative and qualitative data collection methods will be applied [16]. Each case study will generate quantitative big data derived from the case study interventions when using sensor devices or mobile phone apps. Furthermore, case study participants’ user experience (UX) regarding their Living Lab experiment will be collected with a survey, including a group of harmonized questions across the case studies (quantitative data). In addition to testing the applicability of the suggested Living Lab and big data collection methods, each case study intervention has a specific health and well-being RQ and data collection method to evaluate either the effects of these interventions or their user acceptance. Each case study is defined more specifically in the intervention section.

#### Phase 3: The Ex Post and Engaging

Phase 3 focuses on collectively evaluating case study experiences from a Living Lab operator's point of view. A cross-case analysis grounded on semistructured interviews will be conducted to identify the challenges and benefits of using the proposed research protocols. The following RQ is defined for this phase:

- RQ3: On the basis of real-life case study experimentation, what are the key challenges and critical success factors for developing big data–driven hybrid personas?

### Recruitment

#### Living Labs and Harmonization Body Recruitment

Regarding Living Lab RI harmonization objectives and RQs (RQ1 to RQ3), the unit of analysis is a Living Lab. The 9 VITALISE project Living Labs form the main sample group. The VITALISE project website provides links to Living Lab hosting organizations’ home pages, where more detailed information about each Living Lab and its infrastructure is available [23]. Each Living Lab will indicate a dedicated contact person who will act as a key informant for RI harmonization–related research activities. Furthermore, this contact person can recruit additional experts from their Living Labs when needed. Thus, depending on the research activity, the number of representatives per Living Lab typically varies between 1 and 5 persons.

The external harmonization body members represent a wider community of Living Lab stakeholders comprising known Living Lab researchers and practitioners, health care professionals, and policy makers beyond the VITALISE project. Personal invitations are sent to persons matching the defined profile (ie, having extensive research, practical, or managerial experience from Living Lab research, projects, or organizations). The harmonization body will comprise approximately 10 to 15 persons. Finally, the open access web-based cocreation event participants will be recruited by open calls distributed via the ENoLL and VITALISE project partner communication channels such as newsletters, social media accounts, and websites.

#### Recruitment for Case Studies

The following eligibility criteria are common in all case studies: (1) willing and interested to collaborate voluntarily in the study and conduct study-specific activities defined in the case study interventions that vary case by case, (2) conserved the ability to understand, (3) felt physically and cognitively able to take part in the particular case study (self-assessed), and (4) signed the consent for participation in the study and data processing (transfer of the collected data to an open access database is optional and does not imply exclusion from the study). Otherwise, the sample sizes, recruitment methods, and participant eligibility criteria vary among the case studies and are defined as part of the case study intervention descriptions.

### Interventions for Phases 1 and 3

In the following sections, the interventions for developing and testing harmonized research protocols for big data–driven hybrid personas are defined based on the Template for Intervention Description and Replication checklist and guidelines [24].

#### Why

Lately, Living Labs have gained increased popularity among scholars and practitioners. Although the Living Lab community is already strong and continues to grow as singular entities, it still fails to provide and function according to unified and harmonized research processes that are easily accessible and exploitable by academia and industry researchers beyond the Living Lab internal researchers. As a result, there is a need to identify and test joint research protocols, methods, tools, and devices that could act as an effective and convenient interface to Living Lab infrastructures in various research settings, including everyday living environments research using big data approaches.

#### What (Materials)

The data collection methods, tools, and devices currently used by Living Labs will be collected via the following 3 different structured Microsoft Excel template files: (1) data collection devices, (2) validated scientific scales and questionnaires, and (3) Living Lab services, including prefilled service and method descriptions derived from the Product Validation in Health project’s final report [3]. Guidelines for filling Microsoft Excel files are provided in a PDF document. The filled Microsoft Excel templates will also be used as background material and interview templates during each Living Lab interview. Summary reports (Microsoft Excel and Microsoft Word files) explaining the data collection summary results will be used to share information among all VITALISE Living Labs. The research protocols for each case study will be written as Microsoft Word documents using a structured format.

#### What (Procedures)

##### Phase 1: The Ex Ante and Engaging

First, by following written guidelines, each Living Lab will self-complete the provided Microsoft Excel files together with their core team members by adding new content or modifying the prefilled content. Second, structured interviews with each Living Lab will be conducted to deepen the understanding of each Living Lab’s answers using the previously filled Microsoft Excel templates as an interview notes template. If certain questions remain unanswered during the interview, Living Labs will be requested to fill the missing information afterward directly to the interview notes template, which includes open questions. Third, 2 facilitated web-based cocreation workshops—1 with VITALISE Living Labs and 1 open access event during the ENoLL web-based conference—will be arranged to collect qualitative feedback and identify new Living Lab methods, tools, and services beyond the Microsoft Excel template lists. Fourth, research protocols for the case studies are cowritten and discussed in a series of web-based workshops and sending of the documents back and forth between the key researchers from each Living Lab.

##### Phase 2: The In Situ and Engaging

The detailed research protocol descriptions for each case study are provided in the follow-up *Case Study Interventions for Phase 2* section.

##### Phase 3: The Ex Post and Engaging

First, semistructured interviews (qualitative data) with VITALISE Living Labs will be conducted to identify the key experiences regarding case studies from a Living Lab operator’s point of view. In the follow-up process, a series of facilitated cocreation workshops will be conducted to define a harmonized research protocol for developing big data–driven hybrid personas based on case study results.

#### Who Provided

Interviews and workshops will be managed and delivered by a small group of experienced Living Lab researchers (ie, 3 persons) taking part in the VITALISE project and having extensive experience in planning and implementing Living Lab research projects and protocols. Support personnel will be involved in the implementation of the study (eg, for note taking).

#### How

VITALISE Living Labs will be first instructed to fill Microsoft Excel file templates by each individual Living Lab team member and then self-arrange a face-to-face or web-based workshop where team members can collectively discuss and refine the Living Lab’s responses. The follow-up Living Lab interviews will be conducted by 1 to 2 interviewers and 1 note taker in Microsoft Teams, Google Meets, or Zoom videoconferencing platform. Notes will be written interactively as the interview progresses, and the videoconference screen-sharing feature will be used to share the written information among all participants. Afterward, Microsoft Teams will be used for document and data sharing among Living Labs during the research process. Mentimeter, an interactive presentation platform, will also be occasionally used for brainstorming during the web-based workshops.

#### Where

Owing to the long distances between the VITALISE Living Labs and the COVID-19 pandemic situation, all interviews and workshops will be arranged on the web using Microsoft Teams, Google Meets, or Zoom videoconferencing platforms. If the COVID-19 pandemic situation allows, face-to-face workshops will be organized as a part of the VITALISE project consortium meetings during phase 3.

#### When and How Much

During phase 1, each Living Lab’s internal workshop where they collectively discuss and refine their existing Living Lab practice responses will occur once and is expected to take approximately 3 hours. If the time is not sufficient, Living Labs will be encouraged to keep as many workshops as required to properly fill the Microsoft Excel templates. Living Lab interviews focusing on deepening the understanding regarding each Living Lab’s current practice will occur once and is expected to last approximately 2 to 2.5 hours. Living Lab interviews that focus on evaluating the key experiences regarding the case studies from the Living Lab operator's point of view will also occur once and are expected to last approximately 2 to 2.5 hours each. It is estimated that at least three (web-based) workshops with an estimated 3-hour duration each are needed to agree on the harmonized research protocols for developing big data–driven personas. After each workshop, a web-based voting procedure will be conducted to evaluate the consensus among Living Labs and harmonization bodies. The proposed research protocols will be modified until the voters reach a supermajority agreement (ie, at least a 70% acceptance rate). If an agreement is not reached during the first 5 iterations, the process will end, and disagreements regarding the research protocol and included technologies, methods, tools, and other scientific instruments will be documented.

#### Tailoring and Modifications

In principle, interviews and workshop interventions follow the same principles for all participants, and modifications are not preplanned. However, if a need to make modifications emerge, they can be made.

### Case Study Interventions for Phase 2

#### Case Study 1: Digital Coaching App Impact on Self-efficacy and Well-being

##### Why

The general aim of this case study is to test the digital coach app of the Austrian Institute of Technology (AIT) by analyzing to what extent it can influence individuals’ self-efficacy and well-being by motivating older adults to practice a healthy lifestyle. Moreover, the study will also assess the system’s usability, learnability, and user acceptance. The monitoring of the effects of the coach app use may throw light on the capability of digital coaching in helping retirees maintain (and improve) social relationships and activities that could be put at risk by their withdrawal from the workplace. The study evaluations will be set up in 2 different sites: Belgium and Austria. This multisite design will allow the evaluation of the AIT coach app in different social and cultural contexts.

##### To Whom (Sample Size and Recruitment)

A total of 40 persons will be recruited via the AIT (Austria) and LiCalab (Belgium) user panel databases. Social media channels and collaborations with local authorities will also be used. Key inclusion criteria include the following: for group A, older employees in good health (maximum approximately 3 years before retirement; work continuity during the study), and for group B, retired older people (retired for no longer than 3 years). For both groups, the availability of smartphones (Android version 8.0 or up, or iOS) was a key inclusion criterion. Key exclusion criteria include age <55 years and middle-to-severe constraints in mobility or cognition (self-assessed). For group B, care dependency (self-assessed) is also an exclusion criterion.

##### What (Materials)

Within the study, AIT’s digital coaching app will be provided to the participants. The software used for the trials comprises a smartphone-based app and a server component providing the content and collecting the data. Communication between the front-end and back-end is established over the internet using secure communication channels. The back-end server runs within the facilities of the AIT’s partner organization, ProSelf, to ensure privacy and security. No personal data will be stored in the cloud. A paper user manual will be provided to the participants to guide them through the first steps of installing, configuring, and using the app. Informed consent will be provided in paper form; questionnaires for collecting quantitative data will either be provided in paper form or as a link to a web-based questionnaire.

##### What (Procedures)

The study will follow a mixed methods design in which qualitative and quantitative data will be collected. To determine the effects of AIT’s digital coaching on the 8-week term, 2 measurements (time point 0 [T0] and time point 1 [T1]) will be conducted using standardized tests. Thus, 2 face-to-face sessions will be scheduled with participants: 1 just before the beginning of the study and 1 at the end, after 8 weeks of use. Both qualitative and quantitative outcomes will be measured. At baseline (T0, before the start of the intervention), demographic data will be collected. The following outcomes will also be measured both at baseline (T0) and after the end of the intervention (T1): patient-reported outcome measures, including health and well-being (Short Form-36 version 2) and social life (Lubben scale), self-efficacy (General Self-Efficacy Scale-6), and patient-reported experience measures including, UX (User Experience Questionnaire [UEQ]) and basic health measures (eg, weight).

##### Who Provided

In this study, a multidisciplinary team comprising UX experts, clinical researchers (psychologists and researchers with experience in social sciences), and support personnel will be involved in the implementation of the study. All members hold relevant experience in EU health projects and eHealth research and are experienced in planning, implementing, and analyzing clinical trials.

##### How

Within the study, AIT’s digital coaching app will be provided to the participants. Participants will be provided with a link to install the app on their own smartphones. The app will be provided individually to all participants in the study.

##### Where

Sample group participants will be using their digital coach mobile app at their private homes in Austria and Belgium. This RI is classified as a combination of distributed (ie, private homes) and virtual (ie, mobile app) facilities.

##### When and How Much

The AIT’s digital coaching app will be provided to the participants over a duration of 8 weeks. For data collection, 2 face-to-face sessions (each approximately 2 hours) will be scheduled with participants: 1 just before the beginning of the study and 1 at the end after 8 weeks of use.

#### Case Study 2: Net Zero Energy Buildings Smart Home Living Lab

##### Why

The aim of this feasibility and acceptability study is to evaluate the impact of physical and cognitive activity performance of older adults in a smart home Living Lab environment using a combined approach of virtual apps and several wearable sensors. This particular small-scale pilot study envisages showing us the benefits of specific types of physical and cognitive exercises combined with Living Lab technological advances. Moreover, the study will also analyze the extent to which it can influence individuals’ self-efficacy and well-being by motivating older adults to practice a healthy lifestyle. The monitoring of the effects of the virtual coach app use may shed light on the capability of digital coaching for helping retirees maintain (and improve) social relationships and activities that could be put at risk by their withdrawal from the workplace. Yoga exercises, on the other hand, will provide evidence of how the body reacts by monitoring parameters such as brain activity and heart rate.

##### To Whom (Sample Size and Recruitment)

A total of 15 persons will be recruited by Aristotle University of Thessaloniki through the collaboration and research community for independent living. Participants comprise cognitively intact healthy older adults aged >60 years, with the absence of dementia, severe cognitive impairment, psychiatric diseases, and severe cardiovascular problems.

##### What (Materials)

Data will be collected using the following wearable sensors: Fitbit Charge 3, Empatica E4, and Muse EEG, resulting in physiological big data such as accelerometer, heart rate, and electrodermal activity during the interventions. Chairs will be used as props during the yoga exercises when needed. The following validated questionnaires will be used: (1) UEQ, (2) System Usability Scale, and (3) Unified Theory of Acceptance and Use of Technology, along with predefined key performance indicators will be used to measure the acceptability and usability levels of the proposed protocols. The EuroQoL 5-dimensional 3-level questionnaire (Greek version) will be used to assess the participants’ quality of life. The filled-in Microsoft Excel templates will also be used as background materials and interview templates during each interview. The research protocols for each case study will be written as word documents using a structured format.

##### What (Procedures)

Before cognitive and physical exercises, focus group sessions with beneficiaries and clinicians will be organized for recruitment purposes and participant profile data and end users’ preferences and needs for the used technologies and interventions will be collected. The cognitive and physical exercises in a smart home Living Lab include the measurement of (1) financial capacity; (2) general cognitive status, memory, executive functions, and visuospatial functions; and (3) measurement of physical activity during virtual coaching and yoga exercises while participants are equipped with wearable sensors. After cognitive and physical exercises, focus group interviews will be conducted to assess participants’ experiences regarding usability and acceptance of the performed activities and technologies.

##### Who Provided

A multidisciplinary team comprising Living Lab experts, health care personnel, and gym assistants will participate in the participant recruitment, exercise design, and safety standard definition. Focus groups and questionnaire administration will be managed by a well-coordinated group of experienced Living Lab researchers, with 4 to 5 people taking part in the VITALISE project and having considerable experience in planning and implementing Living Lab research projects and protocols.

##### How

Scheduled visits into a smart home Living Lab will be planned for each participant. All participants will attend all cognitive and physical exercises led by a virtual instructor (exercises 1-3) or a person (exercise 4) in the following order while wearing the Fitbit Charge 3 and Empatica E4 wristband sensors: (1) assessment of participants’ financial capacity using tailored simulated Banking App [25], (2) evaluation of their general cognitive status, memory, executive functions, and visuospatial functions through the Virtual Supermarket app [26]—a simple virtual reality task running on tablet devices or PC for differentiating between MCI patients and healthy older adults, (3) implementation of the virtual coach application [27] for physical activity promotion and (4) applying a yoga session where participants will also wear the Muse EEG during the “body scanning” and “relaxation exercises” as part of the yoga protocol to obtain brain activity during these particular states. After cognitive and physical exercises, focus groups interviews will be conducted to assess participants’ experiences regarding usability and acceptance of the performed activities and technologies.

##### Where

Cognitive and physical exercise interventions will take place in the smart home Living Lab environment equipped with various technologies described previously. Face-to-face focus group sessions will be organized in meeting rooms for vaccinated individuals, whereas for the others, meetings will take place on web-based platforms such as Google Meets or Zoom videoconference.

##### When and How Much

Scheduled visits will be planned for each participant to visit a smart home Living Lab during the spring to summer of 2022. The cognitive and physical exercises will be conducted once, and their durations will be as follows: (1) simulated banking exercise for 5 minutes, (2) virtual supermarket exercise for 30 minutes, (3) virtual physical exercises coaching for 10 minutes, and (4) yoga sessions at a slow but gradually increasing pace, including breathing practice for 10 minutes, chair poses for 15 to 20 minutes, standing poses 10 to 15 minutes, floor poses 15 minutes, and a supine resting pose (Shavasana) for 10 minutes.

##### Tailoring

The interventions will be tailored for all 20 participants. Furthermore, the virtual app used during the interventions will be used in personalized mode to bring innovation.

#### Case Study 3: UX With Gradior Active for Physical Training Reinforcing Dual Task Performance

##### Why

The general aim of this case study is to test the Gradior Active prototype by assessing its usability, learnability, acceptance, and the overall experience of participants, especially in terms of how easy and pleasing it is to use or its perceived utility when embedded in active and healthy aging programs. Collection of UX during an 8-week program implementing the current Gradior Active prototype will be of most importance for the second objective, which is focused on performing a cocreation sprint to understand the challenges for optimizing the program and its implementation in real settings and services (eg, exploring ideal workflows). Ultimately, the case study results will be used to understand what is most important for the UX perspective and for developing big data–driven hybrid personas through the initially described harmonized research protocol.

##### To Whom (Sample Size and Recruitment)

A total of 30 older adults will be recruited through the INTRAS memory clinic and neuropsychological rehabilitation center customers. INTRAS has its own pre-existing testing panel of patients and participants in the active and healthy aging programs, as well as established groups of experts by experience (groups of older adults who, considering their motivations, participate in user-driven design and innovation processes). Participation will be voluntary and will follow best practices in terms of clear information in the information sheet and procedure and informed consent, accomplishing the General Data Protection Regulation, national regulations, and cognitive accessibility parameters. Participants will pertain to 2 groups of older adults aged >65 years: group A, comprising older adults with subjective complaints (for primary prevention), and group B, comprising older adults with mild physical impairment. Exclusion criteria will include participants with sensory or physical impairments that make it very difficult to use the devices (significant hearing or visual impairments) or any nutritional disorder, psychiatric conditions, or neurological problems that make the person unable to participate in the study.

##### What (Materials)

In this study, Gradior Active will be provided to the participants. This is a system for physical and cognitive training that combines motion and cognitive activities with independent and unrelated purposes (*Dual Task*). It incorporates controlled physical exercises (aerobic, strength, balance, and flexibility exercises) using motion capture technologies (Microsoft Kinect). The software comprises a Kinect v2 (Microsoft), a computer and a good size screen or projector, and a server component providing the content and collecting the data.

The program provides step-by-step instructions for the participants, and the sessions will be performed with the support of an experienced professional (eg, clinical psychologist or neuropsychologist). In total, 2 to 3 units will be installed and configured in the INTRAS clinic facilities with the information technology team support. Informed consent will be provided in paper form; questionnaires for collecting quantitative data and UX diaries will be provided either in paper form or as a link to a web-based questionnaire. During the cocreation sessions, easy-to-use digital tools such as Kahoot! (Kahoot ASA) to facilitate social interaction and pleasing moments in the group will also be considered.

##### What (Procedures)

The study follows a mixed methods design in which qualitative and quantitative data will be collected. To determine the effects of Gradior Active on the 8-week term, 2 measurements (T0 and T1) will be conducted using standardized tests. Thus, 2 face-to-face sessions will be scheduled with participants: 1 just before the beginning of the study and 1 at the end after 8 weeks of use. At baseline (T0, before the start of the intervention), demographic data will be collected. The following outcomes will also be measured both at baseline (T0) and after the end of the intervention (T1): patient-reported outcome measures, including health and well-being (Short Form-36 version 2), social life (Lubben scale), Geriatric Depression Scale (GDS), Physical Status Scale (Rapid Assessment of Physical Activity), and (4) Borg Scale of Perceived Exertion; and patient-reported experience measures, including UX (UEQ) and usability and acceptance (System Usability Scale).

##### Who Provided

INTRAS counts with a multidisciplinary team comprising clinical researchers and technologists widely familiar with UX research and studies with digital health and well-being solutions and experts in the participatory methodologies that will be involved in the implementation of the study. All members hold relevant experience in EU health projects, eHealth research, and planning, implementation, and analysis of clinical trials. The study will be coordinated by a psychologist (panel manager), neuropsychologist (scientific coordinator), and gerontologist (project manager) with >10 years of experience in clinical services, (EU) health projects, eHealth research, user research, Living Lab research, and implementation and analysis of real-life tests.

##### How

The study evaluations will be set up in a single site in Spain with a quasi-experimental format without a control group, where each participant acts as their own control through the pre- and postdesign. The study is divided into 2 phases to accomplish the indicated aims. The first phase collects UX with the Gradior Active prototype during the 8-week testing period. The second phase will be focused on a cocreation sprint bringing the individual experience to a group discussion to understand what is linked to perceived value and opportunities for improvement based on value-driven brainstorming exercises (from the current prototype to ideal service offer). These interactive group sessions will include icebreakers, co-design methods, and adequate tools for the purposes. Testing sessions with Gradior Active will be performed individually and in a face-to-face format in the facilities of the Living Lab and INTRAS Clinic. The sessions will be supervised by an experienced therapist. Cocreation sessions will be conducted face to face and in groups. The sessions will take place with the support of 2 experienced facilitators in the INTRAS facilities. For both the stages and planned activities, COVID-19 safety protocols will be in place.

##### Where

A case study will be conducted in the INTRAS rehabilitation Living Lab in the neuropsychological rehabilitation center (with access to INTRAS care centers and memory clinics as ecologically valid environments for Living Lab activities).

##### When and How Much

In the first phase of the study, Gradior Active will be provided to the participants over a duration of 8 weeks, and participants will be oriented to perform 2 weekly sessions of 30 minutes under the supervision of experienced professionals. After the end of the intervention period, the same participants who previously tested the prototype will be invited to the 2 cocreation sessions (duration: 120 minutes).

##### Tailoring

Both the prototype testing phase and cocreation phase will be coordinated considering the participant’s availability and other relevant conditions to participate (eg, transport logistics to attend the sessions). Testing sessions with the Gradior Cognitive intervention program will be supervised by an experienced professional who can intervene to provide support as required. In principle, pre- and postinterviews with assessment and workshop interventions follow the same principles for all participants, and modifications are not preplanned. However, if a need to make modifications emerge, they can be made.

#### Cross-country Case Study 4: Multidimensional Physical Fitness Testing Protocol for Aging Persons

##### Why

With older adults, functional independence is directly dependent on physical fitness. Aging is inevitably associated with the declining functions of systems and organs (heart, lungs, blood vessels, and skeletal muscles) that determine physical fitness. However, aging is a complex process involving many variables that interact with one another, for example, lifestyle factors, chronic diseases, and genetics. Relatively little is known about the physical fitness levels needed to maintain physical independence. Many studies have shown that measurements of physical performance can predict future mobility disability, institutionalization, and mortality. Studies have also shown a strong association between measurements of physical performance and self-reported mobility disability. However, most of these prospective studies used only a few measures of physical performance or were targeted at functionally limited persons; therefore, assessments have only limited value for counseling. Therefore, this case study will develop and test a physical fitness testing protocol for older adults in real-life settings in 3 countries: Finland, Spain, and Hungary.

##### To Whom (Sample Size and Recruitment)

In Finland, the sample group will comprise older adults aged >67 years. To recruit older adults with varying physical fitness levels, the following strategy will be applied: open calls to promote free of charge physical fitness level measurement as part of a research project will be published in local newspapers and social media channels in collaboration with the local city administration responsible for older adult services. Furthermore, the collaboration will be conducted with selected nursing home administrators to recruit participants with weaker physical fitness levels directly from nursing homes. It is estimated that approximately 200 to 400 persons will be recruited via open calls and approximately 50 persons from nursing homes. In Hungary, the sample group will comprise people aged >55 years. Social media, posters in outdoor trails and fitness parks, former contact lists, and snowball sampling will be used to recruit the participants. It is estimated that 30 to 50 persons will be recruited; however, because of dropouts during the training program, only 20 are expected to take part in the posttest. In Spain, the main target sample will be men and women aged between 60 and 80 years. of Participants will be recruited in collaboration with the city council of Gernika-Lumo, local nursing homes, and retiree associations. It is estimated that 20 to 40 people will be recruited from all collaborative organizations.

##### What (Materials)

Each participant will have to fill in an informed consent form before any measurements. Participant consent forms are tailored to meet the General Data Protection Regulation standards and local ethical requirements in Finland, Spain, and Hungary. A total of 2 similar score sheets will be created for the physical fitness measurements, 1 for the participant and 1 for the researchers. General reference values and the interpretation of the results will be presented after the measurement session. This material will also be displayed on the webpages for the participant to view later. Participants will also fill a self-completion quality of life questionnaire (World Health Organization Quality-of-Life) before the test and UEQ at the end of the test. The security of the participants in the event will be carefully designed and considered. A security plan has been developed and will be gone through for all participating event staff members. For the assessment or measurement, simple materials will be used, including stop watches, tape measures, chairs, hand weights (3 kg and 5 kg), and cones.

Approximately 40 participants in Finland and all participants in Spain and Hungary will receive armband sensor devices (SenseWear Armband; BodyMedia Inc), which will measure their physical activity levels on a daily basis for 24 hours. During the measurement period, participants will also be keeping a diary. In Finland, other equipment will include a Jamar hand dynamometer (JWL Instruments), Tanita MC-780MA body composition analyzer, peak expiratory flow measurements (Mini Wright; Clement Clarke International), and a standout smart balance board (Smartifier Ltd). In Hungary, participants will receive information regarding the 8-week training program and related relaxation exercises. The training program uses a 1.5 km outdoor fitness trail with 19 physical exercise stations equipped with information boards, which show different variations in how to use the stations and how to get to the next station. In Spain, the Standout Balance Board and Xiaomi Miband 5 will also be used. A small group of participants will receive a smart wristband watch (Galaxy Watch 3), which measures the average daily activity performed on a 4-day basis. The training program will be based on recreational football playing.

##### What (Procedures)

The following physical fitness components will be covered in the physical fitness test in Finland and Spain: cardiorespiratory endurance, muscle strength and endurance, balance, agility, speed, flexibility, and body composition. After signing the consent form and filling the quality of life questionnaire (World Health Organization Quality-of-Life Scale), the participants will go through the following measurement points step by step in selected locations (ie, sports halls, nursing homes, fitness parks, and outdoor facilities): (1) 6-minute walk test or nonexercise cardiorespiratory fitness test, (2) handgrip strength test, (3) 30-second arm curl test, (4) 30-second chair stand test, (5) Smartifier balance board test, (6) 8-foot up-and-go test, (7) backward walk test, (8) 10 m walk test, (9) chair sit-and-reach test, (10) back scratch test, (11) height and body composition measurements, and (12) peak expiratory flow measurement. In Hungary, which is used as a control group, physical fitness will be measured by following a protocol defined in the senior fitness test manual [28]. After finishing the measurement track, participants will fill the UEQ, which also includes an open text box to propose improvement suggestions or give complaints about the testing procedure.

In Hungary and Spain, the physical fitness testing battery will be used to determine the baseline (T0, before the start of the training program intervention) and conduct the postmeasure (T1) at the end of the 8-week training program. Participants’ daily activities will also be measured with the armband device for 4 days during the training period. In addition, in Spain, some of the participants will also use a smart wristband watch (Galaxy Watch 3). In Hungary, the training program will include outdoor training on fitness trails with 19 stations in a 1.5 km route. Feedback will be collected from participants after the training intervention. In Spain, outdoor training on recreational football will be performed in a network of distributed resources and public spaces. Feedback will be collected from participants after the training intervention.

##### Who Provided

In Finland, physiotherapy students will collect the materials under the supervision of experienced physiotherapy teachers and VITALISE project team members. The testing protocol will be introduced and trained for the students on a separate occasion before the measurement events. VITALISE project team members have extensive experience in planning and implementing Living Lab research projects and protocols in health and well-being research settings. In Hungary, Trebag staff will assist in the data collection of physical fitness tests with the guidance of an experienced and qualified leader. Outdoor fitness occasions will be facilitated by qualified coaches. In Spain, the Gaia staff will assist in data collection of the physical fitness tests, together with students of science degree in physical activity and sport from Deusto University, who will also assist in the fitness occasions.

##### How

During the measurement day in Finland and Spain, there will be an open access period when measurements will be conducted by following the walk-in principle. Participants will circulate individually from the measurement point to another, where they will receive instructions from students (physiotherapy or similar). After finishing the measurement track, participants will go to the meeting point where general reference values and the interpretation of the results can be self-read from the presented materials. At the meeting point, participants will also fill the UEQ questionnaire. In Hungary, the process will be similar; however, participants will be invited, and the measurement track will be based on a protocol defined in the senior fitness test manual [28].

The physical fitness testing protocol will be first run in Finland. Following this, modifications will be made to the testing protocol if needs for change are identified based on participant feedback. The same or revised protocol will then be used in Spain to collect cross-country data. In Hungary, a different protocol [28] will be used to get the baseline for UX and be able to compare how the extended measurement protocol influences the UX. At the end of the project, result comparisons regarding measurement day experiences will be made between the 3 countries and 2 different measurement protocols.

The participants receiving the armband device will be invited to a specific location (eg, university campus), where they will receive detailed information about the test and how to keep the activity diary. In Spain, some of the participants will also receive a Galaxy Watch 3 device, which is used for similar purposes as the armband device. In Finland, participants will keep the device for 1 day (24 hours). In Hungary and Spain, participants will keep it for 4 days (96 hours). Owing to the limited number of devices and device rotation, the measurement time will vary between participants.

In Hungary, outdoor training on fitness trails will be introduced by guided visits where participants can try to experience fitness station activities with the help of a qualified trainer. In the fitness stations, participants will perform exercises with their own body weight. At the stations, boards will show the planned, recommended tasks that participants can perform by 3 variants and how to get to the next station. Feedback collection and postmeasurement (T1) will be conducted after 8 weeks of training interventions.

In Spain, outdoor training on recreational football will be performed in a network of distributed resources and public spaces. Owing to possible COVID-19 restrictions, web-based sessions will be organized if physical restrictions are in place, following 2 methodologies if needed: through live sessions with the instructors and through tutorials so the users could watch them at any time, which will be available on the Gaia Cluster YouTube account. For group web-based exercises (where users are at their homes), WhatsApp group video calls will be used. In these calls, the monitors will bring the classes home and give instructions so that the participants can feel part of the group again. Feedback collection and postmeasurement (T1) will be conducted after 8 weeks of training interventions.

##### Where

In Finland, the open access measurement day will be arranged in a big sports hall and in a few nursing homes. Participants participating in 24-hour measurements will perform their normal daily activities; thus, the location can cover home and various indoor and outdoor environments. In Hungary, the premeasurement day will take place at the Trebag premises and the Rákospalota Elderly Centre. The 8-week training program will take place at protected natural parks in Budapest, which are equipped with various fitness equipment. In Spain, the open access measurement day will be arranged in a public space in the Gernika-Lumo municipality and, possibly, in a nursing home as well. Participants participating in the 96-hour measurements will perform their normal daily activities; thus, the location can cover home and various indoor and outdoor environments.

##### When and How Much

The first measurement events in each country will happen for 1 or 2 days in spring to summer of 2022 at a spacious venue (eg, sports hall, gym, or similar space having enough space to establish the measurement track). Each participant will complete the measurement track once, which will take approximately 20 to 25 minutes in Finland and Spain and 15 to 20 minutes in Hungary. In Finland, the main measurement events will take place first, and smaller measurement events in nursing homes will take place soon after the main measurement session. Participants with armband sensor devices will keep the device for 1 day. In Hungary, after the measurement event, the outdoor fitness training program will start, and it will last for 8 weeks. Participants with armband sensor devices will keep it for 4 days. In Spain, outdoor physical interventions will last for 8 weeks after the measurement event.

##### Tailoring

Participants are expected to participate in all measurement points and preplanned activities; however, they can choose to take part in only a selected number of tests and activities, as participation will be voluntary. The physical fitness testing protocol will be first run in Finland. Modifications will be made to the testing protocol if needs for change are identified based on participant feedback. Thus, it is possible that, in Spain, a slightly modified version of the measurement track will be used. However, for each study participant, the track will be identical.

#### Cross-country Case Study 5: Characterizing the Interaction of the Effects of a Museum Visit on Mobility, Cognition, and Well-being in People With Stroke

##### Why

Although there are studies that have explored the impact of a museum experience on healthy older adults or individuals with acquired and degenerative neurological conditions [29-31], there is a lack of studies investigating the interaction of the effects of a museum visit on mobility, cognition, and the lived experience of individuals with stroke, as well as on older healthy aging adults using advanced technologies that measure mobility, cognition, well-being, and UX. To this end, this small-scale pilot aims to obtain measurements of mobility, cognition, and well-being of people with stroke and healthy aging individuals using innovative technologies to explore the lived experiences of these individuals as they participate in a museum environment. The collected data will be used to (1) study the feasibility of conducting a series of diverse measurements in a museum setting, (2) study the acceptability of such interventions in a museum setting, and (3) create a user model that can be adopted to describe people with disabilities visiting a museum in future big data collection.

##### To Whom (Sample Size and Recruitment)

In Canada, a total of 30 persons will be recruited via a patient contact list from a rehabilitation hospital and the Association Québécoise Des Personnes Aphasiques. In Greece, a total of 20 persons will be recruited via collaboration with the B Department of Neurology, AHEPA University Hospital. All participants will be aged between 55 and 85 years, have normal or corrected vision and hearing, and walk independently with or without technical aids. Group A will comprise 15 persons with stroke, including 6 with mild or mild to moderate aphasia in Canada and 10 people with stroke, including 4 with mild or mild to moderate aphasia in Greece. Group B will comprise 15 healthy controls for Canada and 10 healthy controls for Greece. For both groups, the key exclusion criteria will include (1) postural and balance disorders, (2) severe psychiatric disorders (excluding depression or anxiety), (3) recent history of alcohol or substance abuse, (4) general anesthesia within the past 6 months, (5) pain >2 on the visual analog scale, and (6) >10 on the GDS. In group A, participants with severe or global aphasia or scoring <20 in a mini mental state examination (MMSE) will be excluded. In group B, participants having a <26 score in MMSE will be excluded.

##### What (Materials)

The physical spaces of the museums (benches, stairs, exhibition halls, and vestibules), works of art (paintings and sculptures, casts, and antiquities), and audio guides will be used in the study. Furthermore, the following technologies will be used both in Greece and in Canada to monitor the participants before, during, and after their visit: (1) Pupil Labs eye-tracking glasses and (2) smartwatch (eg, Apple Watch or Fitbit 4). In Canada, additional materials will be used for monitoring, namely the (1) near-infrared spectroscopy (NIRS) system, (2) pressure sensitive walkway (Gaitrite), (3) portable force platform to measure body weight distribution and center of pressure oscillations, and (4) APDM inertial sensors placed in the participants’ thorax and tibia. Physical standardized test alternatives will be used in Greece to gather similar information. Specifically, the physical test battery will include (1) 10 m walk test, (2) Tinetti test, (3) Berg Balance Scale, (4) 30-inch chair stand test, (5) Timed Up and Go test, (6) 2-minute walk test, and (7) balance on 1 leg test. The following test batteries will be used for previsit profiling: (1) for cognitive status measurement, the MMSE or Montreal Cognitive Assessment, and (2) GDS. A neuropsychological evaluation will also be conducted before each session, specifically the (1), Rey Auditory Verbal Learning Test, Stroop Test, and Trail Making Test A+B; (2) Warwick–Edinburgh Mental Well-being Scale; (3) State-Trait Anxiety Inventory and visual analog stress; (4) Visual Analog Pain Scale; (5) Multidimensional Fatigue Inventory; (6) EuroQol-5 dimensions; and (7) Environment Quality and Satisfaction Tool.

##### What (Procedures)

The study will run in 2 sessions (session 1 and session 2), and session 1 will comprise 2 options based on the participants’ profiles. The procedures that will be followed in each session are described below.

In session 1 (option A; participants with stroke and aphasia and half of the control participants), after signing the consent form, participants will be asked to wear the tracking and monitoring equipment (smartwatch, eye-tracking glasses, and headphones for audio guide). For each of the 3 chosen works of art, the participants will listen to the description of the work of art (audio version of the written information that accompanies the work), then listen to the adapted description, and answer some questions posed by the on-site assistant who will assess their experience.

For session 1, option B (participants with stroke and no aphasia and half of the control participants), after signing the consent form, participants will be asked to wear the tracking and monitoring equipment (smartwatch and NIRS cap only for Canadian participants). The participants of this group will be asked to walk twice through a predetermined circuit of 6 different works of art (painting or antiquities) and complete a guided tour. In each painting, participants will be instructed to (1) analyze each painting by focusing on the message or intention communicated by the artist, the symbolic meaning, and the composition of the artwork (ie, analytical condition); and (2) view the painting without analysis (ie, control condition).

In session 2 (all participants), participants will be asked to wear the tracking and monitoring equipment (smartwatch) and walk at their own pace on the predetermined tour of the spaces and the built environment of the museum (stairs, elevator, bench, chair, and toilet). Breaks will be provided as needed and requested by participants.

In both data collection sessions, the lived experience of individuals, as well as their opinions regarding the evaluations at the museum, will also be documented to understand the lived experience of all individuals involved.

##### Who Provided

Researchers from the study, as well as trained research assistants or students, will be present for each session at the museum and will be responsible for the placement of the various forms of technology on the participants and for all data collection.

##### How

All sessions will be held face to face, and only 1 participant will be tested at a time. After signing the consent form and completing the previsit testing batteries with a research assistant in a reception room, the participant and their caregiver or companion will go to the museum exhibit hall. Before the museum experience, participants will be equipped with the following devices: in session 1 option A, (1) smartwatch (Apple Watch or Fitbit), (2) headphones for the audio guide, and (3) eye-tracking glasses; in session 1 option B, (1) the NIRS cap for Canadian participants and (2) a smartwatch (Apple Watch or Fitbit); and in session 2, a smartwatch. In each session after the museum visit and the performed activities, the devices will be removed, and participants will return to the reception room, where a brief questionnaire Will be administered. The participants will receive a smartwatch that they will keep until they return in the following week for session 2.

##### Where

All testing in Canada will be performed at the Montreal Museum of Fine Art, Pavilion for Peace. In Greece, testing will be conducted at the Museum of Casts and Antiquities, School of Philosophy of the Aristotle University of Thessaloniki, Greece.

##### When and How Much

The measurement events in each country will happen for 1 or 2 days in spring to summer of 2022, with a 1-week period between sessions. Session 1 will last a total of approximately 1.5 hours, and session 2 will last approximately a total of 1 hour.

### Analysis

#### During Phases 1 and 3

Inductive qualitative content analysis will be conducted to answer RQ1 to RQ3. First, an iterative open coding process will be conducted, and preliminary categorization will be generated to classify the raw data received from the Microsoft Excel file templates, interviews, workshops, and Mentimeter brainstorming. Next, the data will be grouped by reducing the number of categories by grouping similar technologies, methods, tools, and validated scientific questionnaires to identify alternative approaches to execute a certain research task. Finally, abstraction will be conducted to define the main categories and create possible subcategories under each main category. The popularity of the different technologies, methods, tools, and validated scientific questionnaires among VITALISE Living Labs will be identified by calculating frequencies and percentages. To evaluate the acceptance and consensus of the defined harmonized research protocols among VITALISE Living Labs and external harmonization bodies, an iterative web-based Delphi process will be conducted until a supermajority agreement is reached (ie, at least a 70% acceptance rate) [32].

#### Data Analysis Plans for Case Studies During Phase 2

The *main quantitative analysis approaches* will include descriptive data (eg, mean, frequency, and SD) to characterize the participants’ (sociodemographic variables) and summarize the results of the questionnaires. Chi-square tests will be used to detect the associations between the categorical variables when needed. To explore whether the outcomes of the interventions are significant, parametric (eg, paired and unpaired 2-tailed *t* tests, Pearson correlation, and 1-way analysis of variance) and nonparametric tests (Wilcoxon signed-rank test, Mann–Whitney *U* test, Spearman correlation, and Kruskal–Wallis test) will be conducted to investigate the possible research findings and future research directions. Case studies evaluating the impact of the intervention will include 2 temporal data collection moments, 1 before and 1 after the intervention period, to validate the changes that might occur. Standardized statistical tools will be used for data analysis, such as SPSS (IBM Corp) and Microsoft Excel. Sensor data analysis will be used by the sensor device–related software features, for example, for eye-tracking data or wearable sensor temporal activity data analysis, when case studies use them for data collection. The main quantitative analysis approaches include the inductive qualitative content analysis executed in phases 1 and 3. For case study 5, hermeneutic analysis of the phenomenon and content analysis of the photos will also be performed. For all cases, answers to the questionnaires will be extracted and triangulated with quantitative data.

### Incentives

The VITALISE project consortium Living Labs will receive EU funding (under grant agreement 101007990; April 2021 to March 2024) for conducting the proposed study. According to the consortium agreement, each Living Lab is obligated to conduct such a study; therefore, funding is tightly interlinked with the delivery of the proposed study. Users’ (ie, study participants) intrinsic and extrinsic motivations are key drivers of Living Lab research [33]. Prior studies have identified the following motivational factors that also create the foundation for the case study incentives [34]: (1) projects improving own health or are close to participants’ health needs or interests; (2) projects helping the wider community and contributing to the common good; (3) contributions to formal acknowledgment instead of financial compensation or recognition by others; (4) knowledge seeking, curiosity, and being entertained; (5) desire to feel competent and self-determined; and (5) the possibility to test new innovative products and services. In addition to the abovementioned motivational factor–driven incentives, the following case study incentives will be provided:

1. Case study 1: Free of charge digital coaching mobile app training program for 8 weeks focusing on improving well-being and social participation in general and how to deal with challenges related to retirement; The monetary fees offered to compensate for the participation costs (eg, traveling) are €20 (US $20.90) to €40 (US $41.80) euros for Austrian participants and €15 (US $15.70) to €20 (US $20.90) for Belgium participants, and internet-connection will be reimbursed on request of participants
2. Case study 2: Free of charge measurement of personal physical fitness level and cognitive capability
3. Case study 3: Free of charge use of new services aimed at improving physical, psychological, and emotional health in 2 weekly 30-minute sessions for 8 weeks
4. Case study 4: Free of charge measurement of personal physical fitness level, including the ability to compare own results with the peer group; for Spain and Hungarian participants, free of charge training program for 8 weeks focusing on improving physical fitness
5. Case study 5: 2 museum tickets

### Data Management and Ethics Statement

The VITALISE project will encapsulate multimodal data collection phases toward the development and evaluation of novel supportive interventions related to everyday living environments. As the individuals who will provide the data are the cornerstone of these data collection phases, special attention will be given to the compliance of the collection processes with ethical regulations and guidelines on research involving human beings, as well as on the safety of the sensors, tools, and devices involved, and the protection of personal and health data. Project deliverable *D1.2 First version of ethics and safety manual* constitutes a reference guide for the VITALISE investigators by reporting on the international, European, and national ethical regulations, device safety standards, and certifications. Deliverables will also include ethics management and data control actions and guidelines adopted by the consortium that will assist and monitor its compliance with the medical research regulations and guidelines. In addition, a thorough consent form template will be included and will be used by all partners according to their specific protocols.

Deliverable D1.2 presents the first plan for the VITALISE project scheduled data collection to be compliant with the reported regulations and guidelines and evaluates potential concerns early to be mitigated effectively through the proper management of ethical and data handling–related issues within each country and between different countries. The D1.2 deliverable will be publicly available after the completion of the European Commission review process (expected publication time will be by the end of 2021). It is highlighted that D1.2 is a living document that will be regularly updated throughout the project, leading to its final version (Deliverable 1.3) on project month 24.

## Results

As of September 30, 2021, project deliverables *D1.2 First version of ethics and safety manual* and *D2.1 Standard Version 1*, presenting the state of the art for the Living Lab services, methods, and tools, have been submitted for the European Commission review process. Both deliverables will be publicly available after completion of the European Commission review process (expected publication time will be by the end of 2021). Both deliverables are living documents that are regularly updated throughout the project, leading to its final version by month 24. At the time of submission, the status of the VITALISE project is active, and materials for national ethics boards are being prepared. After receiving acceptance from ethics boards, participant recruitment for case studies will be started latest by June 2022. The study will be finished by March 2024.

## Discussion

### Harmonization Process Outcomes

The outcomes of this study enable Living Labs to exploit similar research protocols, devices, hardware, and software for interventions and complex data collection purposes when developing big data–driven hybrid personas. The harmonized protocols will speed up and improve the research quality and offer novel possibilities for open data sharing, multidisciplinary research, and comparative studies beyond current practices. Researchers will have wider, simplified, and more efficient access and services to Living Lab RIs, irrespective of location. This enables researchers to focus on the research itself instead of managing practical challenges. Economies of scale and improved use of resources across Living Labs have also been realized because of the less duplication of services and common development and optimization of operations.

### Case Study Outcomes

#### Case Study 1

This study will allow a better understanding of how digital coaching can influence individuals’ well-being and social participation by motivating older adults to practice a healthy lifestyle. Monitoring the effects of AIT’s digital coaching app use may shed light on the capability of a digital coach for helping retirees maintain (and improve) social relationships and activities that could be put at risk by their withdrawal from the workplace. In addition, the results will also allow the optimization of the UX of deployed coaching mobile apps. By setting up this case study at 2 different sites, Belgium and Austria, specific results related to these different social and cultural contexts are also expected.

#### Case Study 2

The results of the randomized controlled trial will show that it is feasible to conduct a randomized controlled trial of yoga in a virtual and sensor-assisted Living Lab environment using the described protocol. Moreover, the collection of novel sensor data from the experiment is expected to provide insightful information about the physical and psychological status of the participants.

#### Case Study 3

The results of this study will allow the analysis of effectiveness, usability, and acceptance of the described Gradior Active intervention. It will state the pillars for evidence-based decision-making on investment in the upgrade and modernization of the prototype; at the same time, it will ensure a final co-design sprint with participants to orient the incremental development plans.

#### Case Study 4

The main results will focus on understanding older adults’ physical fitness levels by defining and testing comprehensive testing patterns in a cross-country setting. Single test results with reference values exist; however, surprisingly, a wider understanding of older adults’ fitness levels is lacking. From prior studies, it is acknowledged that the vast majority of the participants recruited via open calls will be persons with good fitness and functioning levels. Therefore, older adults with lower physical fitness will be recruited from nursing homes to also cover more fragile and less fit older adults. By combining the daily physical activity level sensor measures and physical fitness test results, novel and insightful information about the physical status of the participant can be verified. The perceived UX of the proposed comprehensive physical fitness level research protocol will be compared with a well-established but less comprehensive protocol. It is expected that there will be no significant differences regarding UX; therefore, in the future, a more comprehensive protocol should be applied to reveal a better understanding of the older adults’ physical fitness levels. Furthermore, the pre- and posttraining period physical fitness levels will be compared in the cases of the Spain and Hungary case studies. These results are expected to reveal that the training program affects participants’ physical fitness level as well as provide suggestions from participants on how to improve the training program.

#### Case Study 5

Although this study is exploratory, it is anticipated that museum visits will be associated with (pre and post) changes in mobility (endurance, navigation, posture, and balance) and cognition (attentional control and auditory comprehension in dual tasking) and will generate a well-being effect (positive experiential experience and decrease in anxiety state). Moreover, considering that museums or any public places solicit locomotor capacities (endurance, navigation, posture, and balance), it is expected that the visit will be associated with a greater variation in motor skill, cognition, and well-being in all our most vulnerable groups (stroke) compared with the control group. Finally, the environmental conditions and characteristics that do or do not influence the above variables will be identified. The latter may result in modifications that will favor inclusion and ultimate participation of aging individuals, especially those living with stroke and language limitations.

## Abbreviations

AIT: Austrian Institute of Technology
ENoLL: European Network of Living Labs
EU: European Union
JRA: joint research activity
MMSE: mini mental state examination
NIRS: near-infrared spectroscopy
RI: research infrastructure
RQ: research question
UEQ: User Experience Questionnaire
UX: user experience
VITALISE: Virtual Health and Wellbeing Living Lab Infrastructure

## References

[1] European Network of Living Labs.

[2] Hossain M, Leminen S, Westerlund M (2019). A systematic review of living lab literature. J Clean Prod.

[3] Santonen T, Julin M, Hirvikoski T, Salmi A, Leskinen J, Saastamoinen K, Kjellson F, Anderson K, Baskyte M, Nigul M (2020). Living lab business models and services key findings from Product Validation in Health (ProVaHealth) project. Laurea-ammattikorkeakoulu.

[4] (2013). Regulation (EU) No 1291/2013 of the European Parliament and of the Council of 11 December 2013 Establishing Horizon 2020 - the Framework Programme for Research and Innovation (2014-2020) and Repealing Decision No 1982/2006/EC. Official Journal of the European Union.

[5] Giffoni F, Vignetti S, Kroll H, Zenker A, Schubert T, Becker E, Ipolyi I, Griniece E, Angelis J (2018). Deliverable 3.1 working note on RI typology. Horizon 2020 Programme.

[6] Jee K, Kim G (2013). Potentiality of big data in the medical sector: focus on how to reshape the healthcare system. Healthc Inform Res.

[7] Jensen PB, Jensen LJ, Brunak S (2012). Mining electronic health records: towards better research applications and clinical care. Nat Rev Genet.

[8] Raghupathi W, Raghupathi V (2014). Big data analytics in healthcare: promise and potential. Health Inf Sci Syst.

[9] Lidwell W, Holden K, Butler J (2010). Universal Principles of Design, Revised and Updated: 125 Ways to Enhance Usability, Influence Perception, Increase Appeal, Make Better Design Decisions, and Teach through Design.

[10] de Heredia A, Goodman-Deane J, Waller S, Clarkson P, Justel D, Iriarte I, Hernández J (2018). Personas for policy-making and healthcare design. Proceedings of the DESIGN 2018 - 15th International Design Conference.

[11] Mulder S, Yaar Z (2006). The User is Always Right: A Practical Guide to Creating and Using Personas for the Web.

[12] Salminen J, Şengün S, Kwak H, Jansen BJ, An J, Jung S, Vieweg S, Harrell DF (2018). From 2,772 segments to five personas: Summarizing a diverse online audience by generating culturally adapted personas. First Monday.

[13] Billestrup J, Stage J, Nielsen L, Hansen K (2014). Persona usage in software development: advantages and obstacles. Proceedings of the 7th International Conference on Advances in Computer-Human Interactions, ACHI 2014.

[14] McGinn J, Kotamraju N (2008). Data-driven persona development. Proceedings of the SIGCHI Conference on Human Factors in Computing Systems.

[15] Leech NL, Onwuegbuzie AJ (2007). A typology of mixed methods research designs. Qual Quant.

[16] Denzin NK (2017). The Research Act: A Theoretical Introduction to Sociological Methods.

[17] Stokes DE (1996). Pasteur™s Quadrant: Basic Science and Technological Innovation.

[18] Lang DJ, Wiek A (2021). Structuring and advancing solution-oriented research for sustainability : this article belongs to Ambio's 50th Anniversary Collection. Theme: solutions-oriented research. Ambio.

[19] Arnkil R, Järvensivu A, Koski P, Piirainen T (2010). Exploring Quadruple Helix Outlining User-oriented Innovation Models.

[20] Schuurman D, De Marez L, Ballon P (2016). The impact of living lab methodology on open innovation contributions and outcomes. Technol Innov Manag Rev.

[21] Lindberg T, Meinel C, Wagner R (2011). Design Thinking: A Fruitful Concept for IT Development?.

[22] Council B (2005). Eleven lessons: managing design in eleven global brands - A study of the design process. Design Council.

[23] The VITALISE-project.

[24] Hoffmann TC, Glasziou PP, Boutron I, Milne R, Perera R, Moher D, Altman DG, Barbour V, Macdonald H, Johnston M, Lamb SE, Dixon-Woods M, McCulloch P, Wyatt JC, Chan A, Michie S (2014). Better reporting of interventions: template for intervention description and replication (TIDieR) checklist and guide. Br Med J.

[25] Stavropoulos TG, Meditskos G, Kompatsiaris I (2017). DemaWare2: integrating sensors, multimedia and semantic analysis for the ambient care of dementia. Pervas Mobile Comput.

[26] Zygouris S, Giakoumis D, Votis K, Doumpoulakis S, Ntovas K, Segkouli S, Karagiannidis C, Tzovaras D, Tsolaki M (2015). Can a virtual reality cognitive training application fulfill a dual role? Using the virtual supermarket cognitive training application as a screening tool for mild cognitive impairment. J Alzheimer's Dis.

[27] Mastoras R, Triantafyllidis A, Giakoumis D, Kordonias R, Papaprodromou A, Nalbadis F, Segkouli S, Lithoxoidou E, Georgiadis C, Paliokas I, Votis K, Tzovaras D (2021). A mobile-based multimodal framework for pervasive health monitoring. Proceedings of the IEEE International Conference on Pervasive Computing and Communications Workshops and other Affiliated Events (PerCom Workshops).

[28] Rikli RE, Jones CJ (2002). Senior fitness test manual. Choice Rev Online.

[29] Mastandrea S, Maricchiolo F, Carrus G, Giovannelli I, Giuliani V, Berardi D (2018). Visits to figurative art museums may lower blood pressure and stress. Arts Health.

[30] Thomson LJ, Lockyer B, Camic PM, Chatterjee HJ (2018). Effects of a museum-based social prescription intervention on quantitative measures of psychological wellbeing in older adults. Perspect Public Health.

[31] Schall A, Tesky VA, Adams A, Pantel J (2018). Art museum-based intervention to promote emotional well-being and improve quality of life in people with dementia: the ARTEMIS project. Dementia (London).

[32] Dalkey N (1969). An experimental study of group opinion: the Delphi method. Futures.

[33] Stahlbrost A, Kareborn BB (2011). Exploring users motivation in innovation communities. Int J Entrep Innov Manag.

[34] Callari T, Moody L, Saunders J, Ward G, Holliday N, Woodley J (2019). Exploring participation needs and motivational requirements when engaging older adults in an emerging living lab. Technol Innov Manag Rev.

